# Validation of the foot length measure as an alternative tool to identify low birth weight and preterm babies in a low-resource setting like Nepal: a cross-sectional study

**DOI:** 10.1186/s12887-015-0361-4

**Published:** 2015-04-17

**Authors:** Ashish KC, Viktoria Nelin, Ravi Vitrakoti, Surabhi Aryal, Mats Målqvist

**Affiliations:** International Maternal and Child Health, Department of Women’s and Children’s Health, Uppsala University, University Hospital, Uppsala, SE-751 85 Sweden; United Nation’s Children’s Fund, Nepal Country Office, UN House, Pulchowk, Nepal; Foundation for Maternal and Child Health Nepal, Kathmandu, Nepal; College of Medical Sciences, Bharatpur, Nepal

**Keywords:** Preterm, Low birth weight, Foot length, Screening tool, Nepal

## Abstract

**Background:**

The majority of infants who die in the neonatal period are born with a low birth weight (LBW, <2500 grams), or prematurely (before 37 weeks). Most deaths among these infants could be prevented with simple, low-cost interventions like kangaroo mother care (KMC) or prevention and early identification of infection. It is difficult, however, to determine birth weight and gestational age in community settings, and therefore necessary to find an appropriate alternative screening tool that can identify LBW and preterm infants.

**Methods:**

This cross-sectional study was conducted at a tertiary hospital in Nepal to compare the validity of using three different foot length measurement methods (plastic ruler, measuring tape, and paper footprint) as screening tools for identifying babies with birth weights <2000 grams or infants born preterm (<37 weeks). LBW was defined as less than 2000 grams because of the implication for use of KMC for these infants. Non-parametric receiver operating characteristics (ROC) analysis was completed to determine which measurement method best predicted LBW and preterm birth. For the method that was the best predictor for each outcome (i.e. highest area under the curve), further analyses were completed to determine sensitivity, specificity, likelihood ratios and predictive values of an operational screening cutoff to predict LBW or preterm birth in this setting.

**Results:**

Of the 811 infants included in this study, 30 infants had LBW and 54 were born preterm. The plastic ruler was the measurement method with the highest area under the curve, and thus predictive score for estimating both outcomes, so operational cutoffs were identified based on this method. An operational cutoff of 7.2 cm was identified to screen for infants weighing <2000 grams at birth (sensitivity: 75.9%, specificity: 90.3%), and 7.8 cm was determined as the operational cutoff to identify preterm infants (sensitivity: 76.9%, specificity: 53.9%).

**Conclusions:**

In Nepal, at least in community settings, foot length measurement with a hard ruler may be a valid proxy to identify at-risk infants when birth weight or gestational age is unavailable. Further studies and piloting should be conducted to identify exact cutoffs that can be used within community settings.

## Background

Of the 135 million live births worldwide in 2010, around 14.9 million infants were born preterm, prior to 37 weeks of gestational age, representing a preterm birth rate of 11.1% [1]. Although this rate varied widely among countries and regions, more than 60% (9.1 million) of all preterm births occurred in sub-Saharan Africa and South Asia, with the preterm birth rate in South Asia being one of the highest at 13.3% of all live births [1]. Additionally, it is estimated that around 13.2 million infants are born with LBW each year, and about half of these births happen in South Asia [2].

The biggest direct cause of neonatal death is complication due to preterm birth, leading to 35% of the 2.6 million neonatal deaths each year globally [3]. Preterm birth is also a significant indirect factor that increases an infant’s risk of dying due to other causes, and further contributes to morbidity among survivors by impairing neurodevelopmental functioning and long-term physical health [4-7]. Although only around 11% of live births result in the delivery of a LBW infant (due to preterm delivery or intra-uterine growth restriction), 60–80% of neonatal deaths are among LBW infants [1,3]. Many of these deaths could be prevented if infants-at-risk were identified early and provided simple interventions like skin-to-skin contact, or kangaroo mother care (KMC); early and exclusive breast-feeding; and prevention and/or early treatment of infections [8,9]. Particularly for these small babies (LBW, preterm, or both), these simple, low-cost interventions could make the difference between life and death in low-resource settings where most deliveries take place at home without the presence of a skilled birth attendant [10-12].

In the community setting, it can be difficult to identify preterm and LBW infants when they are born due to the absence of weighing scales, accurate gestational age measurement, or skilled health workers to assess the newborn’s condition. In fact, birth weight is assessed for only about half of the infants born in these settings, and gestational age is known for even fewer [13]. Previous studies have examined the use of alternative anthropometric measures to identify LBW or preterm babies including mid-upper arm circumference and chest circumference [14-16]. In four recent studies (published in the last 10 years), foot length has been identified as a particularly useful screening tool to identify LBW and/or preterm infants in the community since it does not require in-depth training or unwrapping of the infant, which could expose the infant to hypothermia [17-20].

In Nepal, a low-income country in Southeast Asia, more than 60% of deliveries still occur at home, and only 36% of babies are weighed at birth [21]. Nepal had a national neonatal mortality rate of 33 per 1,000 live births in 2011 and nearly one third of all neonatal deaths were caused by preterm-related complications [2,21]. Therefore, it is important to identify alternative methods to identify those infants at the greatest risk for neonatal death in this setting [3]. A previous study assessing such potential methods was completed in Nepal; however, this study compared the use of chest circumference and foot length (measured with a vertical rule) as screening tools for identifying LBW babies, and did not look at preterm birth as an outcome or additional methods for foot length measurement [17].

The aim of this study was to assess the use of foot length measurement as a screening tool to identify LBW or preterm infants in a hospital setting in Nepal. Three different low-cost tools, requiring minimal training, to measure newborn foot length were evaluated in order to determine an operational cutoff that could be used to screen infants as follows: (1) infants weighing less than 2000 grams or (2) infants with a gestational age less than 37 weeks.

## Methods

### Study setting

This study was conducted at a tertiary, government-run maternity hospital in Kathmandu, Nepal that serves as a central referral hospital. It is also used as a training site for reproductive and neonatal health for the country. The hospital is equipped with 415 inpatient beds, and between 2012–2013 there were 18,132 deliveries at the hospital [22].

### Study design

This was a cross-sectional study examining and comparing the validity of the use of three different foot length measurement methods as screening tools to identify LBW or preterm infants. The validity of these tools was determined based on their comparison to the gold standards of birth weight measured with a scale and gestational age based on last menstrual period. We used the Standards for the Reporting of Diagnostic accuracy studies (STARD) checklist for reporting of the diagnostic accuracy of this study [23].

### Study population

Our study was a subset of a larger study evaluating the impact of the implementation of a simplified neonatal resuscitation protocol on perinatal outcomes at the hospital. For the larger study, a reference population was created to assess the change in perinatal outcomes over a set period of time [24]. This population included a random selection of 20% of women delivering in the hospital. For the purpose of this study, all live born infants from the reference population were selected. The inclusion criteria for this study were all live birth babies whose weight was taken after birth and had birth weight equal to or more than 1000 grams and gestational age of equal to or more than 28 weeks.

Exclusion criteria included multiple births and infants with any of the following conditions: severe respiratory distress (oxygenation support required), severe birth asphyxia (Apgar score <3 at 5 minutes), extreme prematurity (gestational age <28 weeks), extremely low birth weight (<1000 grams), and congenital abnormalities (major or lethal abnormalities, e.g. neural tube or cardiac defect). Furthermore, if parental consent for foot length measurement was not provided, infants were also excluded.

### Sampling technique

This study was conducted from January 1 to March 30, 2014. During this time, 20% of the women admitted in the hospital for delivery were randomly selected using a lottery technique. Specifically, an opaque jar with 100 balls was kept in the admission unit, of which 80 were white and 20 were yellow. For each admission, a ball was drawn from the opaque jar; if a yellow ball was drawn, the woman was enrolled into the study as part of the referent population. The woman was then tracked until delivery, and if the infant was born alive, the mother-infant pair were included as study participants.

### Data collection

In order to complete sampling and data collection, a surveillance team was formed under the close supervision of a research manager (RV). The team consisted of nine female surveillance officers with an academic background in nurse-midwifery and sufficient experience in clinical research to complete round-the-clock surveillance. Four surveillance officers were stationed in the admission unit to conduct the sampling of the referent population and four others in the delivery room to measure birth weight and assess the gestational age. At least one of these surveillance officers was present in these units at all times of day. Finally, one surveillance officer was placed in the postnatal ward during the day shift to measure foot length using each of the three different measurement tools. Surveillance officers were trained in the use of the random sampling technique, weighing of the babies immediately after birth, and foot length measurement methods. Training was completed using demonstration and practice with a skill checklist for 15 infants. RV closely supervised the surveillance officers and reassessed the foot length of each baby in the postnatal ward using all three techniques and provided constructive feedback.

Study participants were weighed using a plastic pan scale with a 50-gram unit of measurement (Narang Medical limited, WS590), which was calibrated at zero before taking the weight of each baby. The gestational age of the babies was estimated from the last menstrual period of the mother. Foot length was assessed using the left foot of the baby and each of the three different measurement tools: a hard plastic ruler, a measuring tape, and by placing the infant’s footprint on a piece of plan white paper. Foot length was measured from the heel to the tip of the big toe in millimeters, for every method. First, the hard plastic ruler was pressed vertically against the babies sole and the reading was done. Then, the baby’s sole was pressed vertically on a hard wooden board and the measuring tape was used to measure the foot length. Finally, the infant’s footprint was taken on a white sheet of paper by using a pencil to mark the tip of the big toe and the heel while keeping the sole of foot vertical on a hard wooden board. The distance between the two marks on the paper, i.e. the infant’s footprint, was then measured using a hard plastic ruler. Surveillance officers were blinded to outcome cutoff values for preterm birth and LBW.

### Definitions

#### Low birth weight

Babies having birth weight less than 2000 grams.

#### Preterm babies

Babies having a gestational age of less than 37 completed weeks at the time of birth.

### Data analysis

Data processing and entry was done using CS-Pro (US Census Bureau and ICF International, Washington DC, USA). The processed data was then analyzed using SPSS Version 17.0 (IBM Corporation, New York, USA). Two binary variables were used to classify birth weight as less than 2000 grams (LBW) or more, and gestational age as less than 37 weeks (preterm birth) or higher. A cutoff of 2000 grams was used for LBW because of the implication for use of KMC to treat these infants. For the remainder of this paper, LBW refers to infants <2000 grams unless otherwise stated. Only infants with outcome measurements according to the gold standards (i.e. known birth weight, measured with a pan scale, and gestational age, according to the date of mother’s last menstrual period) were included in the analysis.

Non-parametric receiver operating characteristics (ROC) analysis was completed individually for each of the foot length measurement tools, and the area under the curve (AUC) was calculated to identify which of the three tools best predicted LBW and preterm birth outcomes. Further analyses were only completed for the method that best predicted LBW and preterm birth, as determined by the AUC. Sensitivity, specificity, likelihood ratios, and predictive values were calculated for a range of foot length measurements so that an operational cutoff could be determined to screen for LBW or preterm infants in the community. For comparison reasons, the cutoff was determined by identifying the foot length with the highest average of sensitivity plus specificity to predict LBW or preterm birth, as this method was used in previous studies. Data are shown as mean ± standard deviation. All differences are considered significant when p <0.05.

### Ethical clearance

The Institutional Review Committee of Paropakar Maternity and Women’s Hospital, Development Board (Ref. no. 55–11 ka-559) and the Nepal Health Research Council (Ref. no. 1191) approved this study. Verbal consent was chosen as minimal risk was involved in the procedure, and was received from the parents of each infant included in the study. The verbal consent form included an easy to understand explanation of the purpose of the study in the local language, a simple description of the procedures, and the duration of the baby’s participation. The verbal consent form was reviewed and approved by the institutional review committee.

## Results

**Figure 1:**
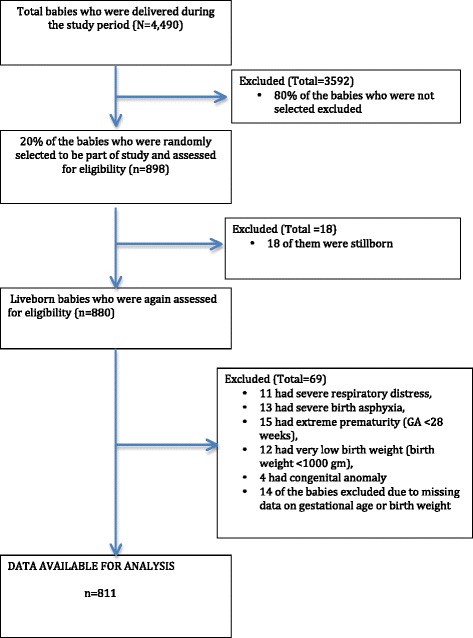
Flow chart of study participants.

During the study period, there were a total of 4,490 women who delivered at the hospital. Twenty percent of this population was randomly selected to be included in the study for foot length measurement, a total of 898 infants. Eighteen (2.0%) infants in the referent population were stillborn and thus excluded from the study population. Further, 55 (6.3%) of the remaining infants were excluded because of exclusion criteria given in the methods section. Fourteen (2.0%) of these infants were also excluded in the final analysis due to missing data, thus the final study population with foot length and outcome measurements included 811 infants (90% of the randomly selected population) (Figure 1).

### Descriptive characteristics

There were 431 male infants (53.1%). The mean birth weight of the infants was 2929 (±532.3) grams, and there were 30 infants who were LBW (3.7%). The mean birth weight of these LBW infants (i.e. weighing between 1000–2000 grams) was 1631 ± 241.6 grams. The mean gestational age of the study population was 39.5 ± 5.7 weeks, and fifty-four of the infants were born preterm (6.7%). The mean gestational age of the preterm infants was 35.0 ± 1.6 weeks.

### Foot length measurements

Mean foot lengths for the various measurement tools were: 7.72 ± 0.02 cm for the plastic ruler, 7.85 ± 0.02 cm for the measurement tape, and 7.65 ± 0.02 cm for paper footprint method (p < 0.001). Table 1 shows the AUC for each of the different measurement tools in predicting LBW or preterm birth. There was no statistically significant difference in the AUC between the three methods for predicting either LBW or preterm birth. The plastic ruler had the highest AUC, and thus predictive score, for estimating both LBW (87.8%) and preterm birth (68.3%). Because the plastic ruler had the highest predictive score, the analysis that follows was only conducted for foot lengths measured with this tool.

**Table 1:** **Area under the curve (AUC) for each of the foot length assessment tools**

	**AUC (%)** ^**a**^	**95% CI**
**Low birth weight (<2000 grams)**		
Plastic scale	87.8	80.1–95.5
Tape	83.6	74.5–92.6
Paper footprint	74.1	64.8–83.4
**Preterm (<37 weeks)**		
Plastic scale	68.3	61.0–75.6
Tape	68.0	60.8–75.2
Paper footprint	59.8	51.8–67.7

^a^AUC was determined using non-parametric receiver operating characteristics analysis.

### Sensitivity, specificity, likelihood ratios and predictive values of foot length measured with plastic ruler

The sensitivity, specificity, likelihood ratios and predictive values of a range of foot length measurements using the plastic ruler are shown in Table 2. The foot length determined to be a potential cutoff for identifying LBW infants using the initial criteria was 7.5 cm. At this foot length, the average of sensitivity and specificity was highest (84.0%), and the positive predictive value (PPV) was 17.3% while the negative predictive value (NPV) was 99.3%. This relatively high cutoff level, with a low PPV due to the low frequency of infants weighing less than 2000 grams at birth, was not considered to be operational, and a second criterion giving higher importance to specificity was calculated. This resulted in a lower cutoff of 7.2 cm, with a PPV of 27.8% and NPV of 99.1%. Within this study population, 110 (13.6%) infants had a foot length below the cutoff of 7.2 cm.

**Table 2:** **Sensitivity, specificity, and predictive values of a range of foot length cutoffs**

**Outcome**	**Foot length (cm)** ^**a**^	**Sensitivity**	**Specificity**	**Initial criterion for cutoff (Specificity + Sensitivity) ½** ^**b**^	**Adjusted criterion for cutoff (Specificity** ^**2**^ **+ Sensitivity) ½** ^**c**^	**LR+**	**LR-**	**PPV**	**NPV**
**Low birth weight (<2000 grams)**	6.7	24.1	99.0	49.1	74.0	24.1	0.8	79.0	97.0
	6.8	24.1	98.9	49.0	73.8	17.2	0.8	73.0	97.0
	6.9	31.0	98.2	53.4	75.8	17.2	0.7	68.0	97.0
	7.0	34.5	97.6	66.1	76.6	14.4	0.7	60.9	97.6
	7.1	72.4	90.8	81.6	84.7	7.9	0.3	28.9	99.0
	7.2	75.9	90.3	83.1	85.5	7.8	0.3	27.8	99.1
	7.3	75.9	88.6	82.3	84.2	6.7	0.3	24.7	99.1
	7.4	79.3	86.8	83.1	84.3	6.0	0.2	22.1	99.2
	7.5	82.8	85.2	84.0	84.4	5.6	0.2	20.2	99.4
	7.6	93.1	60.1	76.6	71.1	2.3	0.1	8.6	99.7
	7.7	93.1	55.9	74.5	68.3	2.1	0.1	7.8	99.7
**Preterm (<37 weeks)**	7.0	17.3	97.4	57.4	70.7	6.7	0.8	32.2	94.3
	7.1	26.9	89.6	58.3	68.7	2.6	0.8	15.6	94.5
	7.2	26.9	88.9	57.9	68.0	2.4	0.8	14.7	94.5
	7.3	26.9	87.1	57.0	67.0	2.1	0.8	12.9	94.4
	7.4	30.8	85.4	58.1	67.2	2.1	0.8	13.1	94.5
	7.5	32.7	83.8	58.3	66.7	2.0	0.8	12.6	94.6
	7.6	67.3	60.4	63.9	62.7	1.7	0.5	10.8	96.3
	7.7	73.1	56.4	64.8	62.0	1.7	0.5	10.7	96.7
	7.8	76.9	53.9	65.4	61.6	1.7	0.4	10.6	97.0
	7.9	78.8	48.8	63.8	58.8	1.5	0.4	9.9	97.0
	8.0	80.8	47.1	64.0	58.4	1.5	0.4	9.8	97.2

^a^Foot lengths measured with hard plastic ruler.

Sensitivity, specificity, average of sensitivity + specificity, likelihood ratios, and predictive values were determined using non-parametric receiver operating characteristics analysis.

^b^Initial criterion for cutoff (Specificity + Sensitivity) ½ is the average of specificity and sensitivity

^c^Adjusted criterion for cutoff (Specificity^2^+ Sensitivity) ½ is the sum of average of square of specificity and sensitivity.

For determining preterm birth, the potential cutoff for foot length identified with the initial criteria was 7.8 cm. At this length, the sensitivity was 76.9% and the specificity was 53.9% (average of 65.4%), and the PPV was 10.6% while the NPV was 97.0%. Using the adjusted criteria, giving more weight to specificity, resulted in a cutoff of 7.0 cm. This increased PPV to 32.2%, but reduced sensitivity drastically to 17.3%. Thus the initial criterion for cutoff was chosen. Within this study population, 390 (48.1%) infants had a foot length below the cutoff of 7.8 cm.

## Discussion

This hospital-based study in Nepal found that in order to predict LBW (<2000 grams) or preterm birth, the plastic ruler method for measuring foot length had the highest predictive score. Within this population, 7.2 cm was identified as the foot length cutoff to predict LBW infants, while a foot length cutoff of 7.8 cm was found to predict preterm birth, when using the plastic ruler as the measurement method. Foot length measured by plastic ruler was a better predictor for LBW than preterm birth with a higher specificity and PPV; however, the PPV for both outcomes was still quite low, likely due to the low prevalence of LBW and preterm birth within this population.

### Strengths and limitations

To our knowledge, this is the first study in South Asia that compares the use of three different foot length measurement methods to identify at-risk infants, including LBW or preterm infants. By comparing the use of these three tools it was possible to identify which method was the most reliable predictor.

There were, however, some limitations within this study. Although a scale was available and the surveillance officers were trained in its use, there was heaping of the infant’s weights around the hundred marks in the measurements, especially at 2,500 grams. There were also some inconsistencies in gestational age within individual mother’s medical records. This may limit the reliability of the outcome data and thus the cutoffs determined. Furthermore, the exclusion of very LBW infants and extremely preterm infants could lead to an underestimation of the sensitivity and specificity, as these infant’s foot lengths would likely have fallen below the determined cutoff.

Additionally, this was a hospital-based study and therefore the rates of preterm birth and LBW may differ from those in the community, our target population. Thus validation for this tool must be completed within in the community among those health workers who will actually be using the measurement tools. Finally, the prevalence of preterm birth and LBW (<2000 grams) were lower in this study population than expected and therefore the PPV for each screening cutoff was low.

### Compared to other studies

The operational cutoff of 7.2 cm to identify LBW (<2000 grams) infants was similar to a previous study from Nepal using a classification for LBW of 2000 grams. This study, from a community setting, found the operational cutoff to be 6.9 cm with a sensitivity of 88% and specificity of 86% [17]. This lower cutoff with higher sensitivity, specificity, and PPV could be due to the larger number of LBW infants in this study population, as well as different criteria for deciding the cutoff [15]. Although these studies were completed in the same country, the first study by Mullany et al. was performed in the community, through home visits. This may also explain the different cutoffs and highlights the need for future studies to be conducted in the community, where the use of this type of screening measurement could be most beneficial. The previous study by Mullany et al. did not explore the use of foot length measurement to identify preterm infants, which is another key area for future research at the community level [17]. The operational cutoff of 7.8 cm for determining preterm infants was similar to those found in other studies (range: 7.5–8.0 cm) [18-20].

### Public health impact

If the adjusted cutoff for LBW (<2000 grams) of 7.2 cm would be used in the community setting, for every 1,000 deliveries, 136 infants would be identified as having a birth weight <2000 grams and receive counseling for extra care practices like KMC, early and exclusive breastfeeding, etc. Of these 136 infants, only 38 would actually weigh less than 2000 grams, while 98 would not have a birth weight less than 2000 grams (this is again likely due to the low prevalence of LBW and thus low PPV). However, even if these infants are included in the program and given additional counseling on care practices, there is no harm done to the infants by receiving these interventions and they can be given at little or no additional cost. Of the 864 infants who would be identified as not weighing less than 2000 grams according to this cutoff, eight infants who actually did would be missed.

If the initial cutoff for preterm birth of 7.8 cm would be used in the community setting, for every 1,000 deliveries, 481 infants would be identified as preterm. Of these infants, 51 would actually be preterm and 430 would not be. Of the 519 infants who would be identified as not preterm, 16 infants who were actually preterm would be missed.

## Conclusions

In Nepal, a country where exact data regarding the prevalence of LBW and preterm birth is not completely known and the majority of deliveries occur at home, foot length measurement as a screening tool has the potential to identify at-risk infants who are in need of additional care. In the community setting, foot length measurement with a hard ruler may be a valid proxy when birth weight or gestational age is unknown. By identifying these at-risk infants early, there is the possibility to provide them with simple interventions like skin-to-skin contact, early and exclusive breastfeeding, and prevention and early treatment of infection, thereby reducing neonatal mortality. Furthermore, even if some infants are falsely identified as LBW or preterm, these interventions will not harm them. Further studies and piloting should be conducted to identify the exact cutoff that can be used in different communities.

## Abbreviations

LBW: Low birth weight
KMC: Kangaroo mother care
STARD: Standards for the Reporting of Diagnostic accuracy studies
ROC: Receiver operating characteristics
AUC: Area under the curve
PPV: Positive predictive value
NPV: Negative predictive value

## References

[1] Blencowe H, Cousens S, Oestergaard MZ, Chou D, Moller AB, Narwal R (2012). National, regional, and worldwide estimates of preterm birth rates in the year 2010 with time trends since 1990 for selected countries: a systematic analysis and implications. Lancet.

[2] Lawn JE, Davidge R, Paul VK, von Xylander S, de Graft Johnson J, Costello A (2013). Born too soon: care for the preterm baby. Reprod Health.

[3] Lawn JE, Blencowe H, Oza S, You D, Lee AC, Waiswa P (2014). Every Newborn: progress, priorities, and potential beyond survival. Lancet.

[4] Lawn JE, Blencowe H, Darmstadt GL, Bhutta ZA (2013). Beyond newborn survival: the world you are born into determines your risk of disability-free survival. Pediatr Res..

[5] Blencowe H, Lee AC, Cousens S, Bahalim A, Narwal R, Zhong N (2013). Preterm birth-associated neurodevelopmental impairment estimates at regional and global levels for 2010. Pediatr Res..

[6] Blencowe H, Vos T, Lee AC, Philips R, Lozano R, Alvarado MR (2013). Estimates of neonatal morbidities and disabilities at regional and global levels for 2010: introduction, methods overview, and relevant findings from the global burden of disease study. Pediatr Res..

[7] Katz J, Lee AC, Kozuki N, Lawn JE, Cousens S, Blencowe H (2013). Mortality risk in preterm and small-for-gestational-age infants in low-income and middle-income countries: a pooled country analysis. Lancet.

[8] Conde-Agudelo A, Diaz-Rossello JL (2014). Kangaroo mother care to reduce morbidity and mortality in low birthweight infants. Cochrane Database Syst Rev..

[9] Bhutta ZA, Das JK, Bahl R, Lawn JE, Salam RA, Paul VK (2014). Can available interventions end preventable deaths in mothers, newborn babies, and stillbirths, and at what cost?. Lancet.

[10] Lawn JE, Kinney MV, Belizan JM, Mason EM, McDougall L, Larson J (2013). Born too soon: accelerating actions for prevention and care of 15 million newborns born too soon. Reprod Health.

[11] Lee AC, Lawn JE, Cousens S, Kumar V, Osrin D, Bhutta ZA (2009). Linking families and facilities for care at birth: what works to avert intrapartum-related deaths?. Int J Gynaecol Obstet.

[12] Lawn JE, Kerber K, Enweronu-Laryea C, Massee BO (2009). Newborn survival in low resource settings–are we delivering?. BJOG..

[13] Lawn JE, Cousens S, Zupan J (2005). Lancet Neonatal Survival Steering Team. 4 million neonatal deaths: when? Where? Why?. Lancet.

[14] Bhargava SK, Ramji S, Kumar A, Mohan M, Marwah J, Sachdev HP (1985). Mid-arm and chest circumferences at birth as predictors of low birth weight and neonatal mortality in the community. Br Med J (Clin Res Ed)..

[15] Sreeramareddy CT, Chuni N, Patil R, Singh D, Shakya B (2008). Anthropometric surrogates to identify low birth weight Nepalese newborns: a hospital-based study. BMC Pediatr..

[16] Raymond EG, Tafari N, Troendle JF, Clemens JD (1994). Development of a practical screening tool to identify preterm, low-birthweight neonates in Ethiopia. Lancet.

[17] Mullany LC, Darmstadt GL, Khatry SK, Leclerq SC, Tielsch JM (2007). Relationship between the surrogate anthropometric measures, foot length and chest circumference and birth weight among newborns of Sarlahi. Nepal Eur J Clin Nutr.

[18] Marchant T, Jaribu J, Penfold S, Tanner M, Armstrong SJ (2010). Measuring newborn foot length to identify small babies in need of extra care: a cross sectional hospital based study with community follow-up in Tanzania. BMC Public Health..

[19] Nabiwemba E, Marchant T, Namazzi G, Kadobera D, Waiswa P (2013). Identifying high-risk babies born in the community using foot length measurement at birth in Uganda. Child Care Health Dev.

[20] Mukherjee S, Roy P, Mitra S, Samanta M, Chatterjee S (2013). Measuring new born foot length to identify small babies in need of extra care: a cross-sectional hospital based study. Iran J Pediatr.

[21] Ministry of Health and Population, New Era, ICF Macro, USAID (2011). Nepal demographic and health survey 2011.

[22] Government of Nepal MoHP, Paropakar Maternity and Women’s Hospital (2013). “Smarika” Annual Report 2012-13.

[23] Bossuyt PM, Reitsma JB, Bruns DE, Gatsonis CA, Glasziou PP, Irwig LM (2003). The STARD statement for reporting studies of diagnostic accuracy: explanation and elaboration. Clin Chem.

[24] Ashish KC, Malqvist M, Wrammert J, Verma S, Aryal DR, Clark R (2012). Implementing a simplified neonatal resuscitation protocol-helping babies breathe at birth (HBB) - at a tertiary level hospital in Nepal for an increased perinatal survival. BMC Pediatr..

